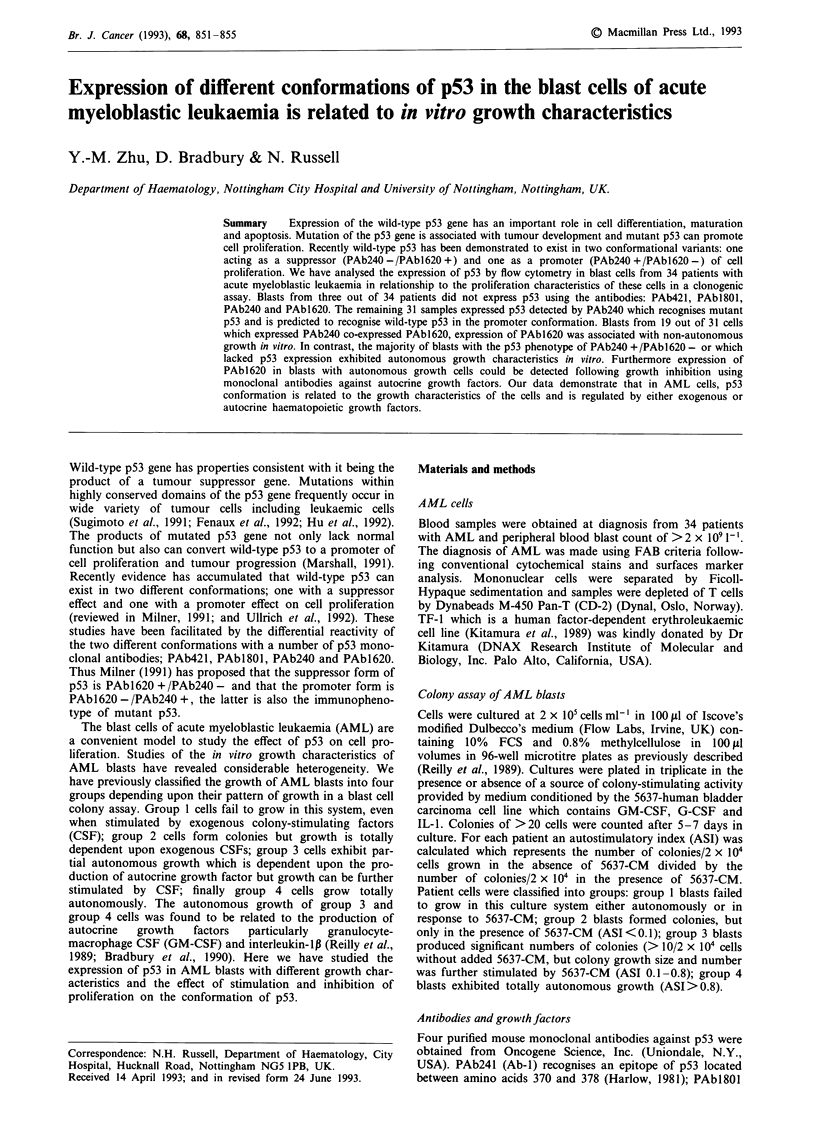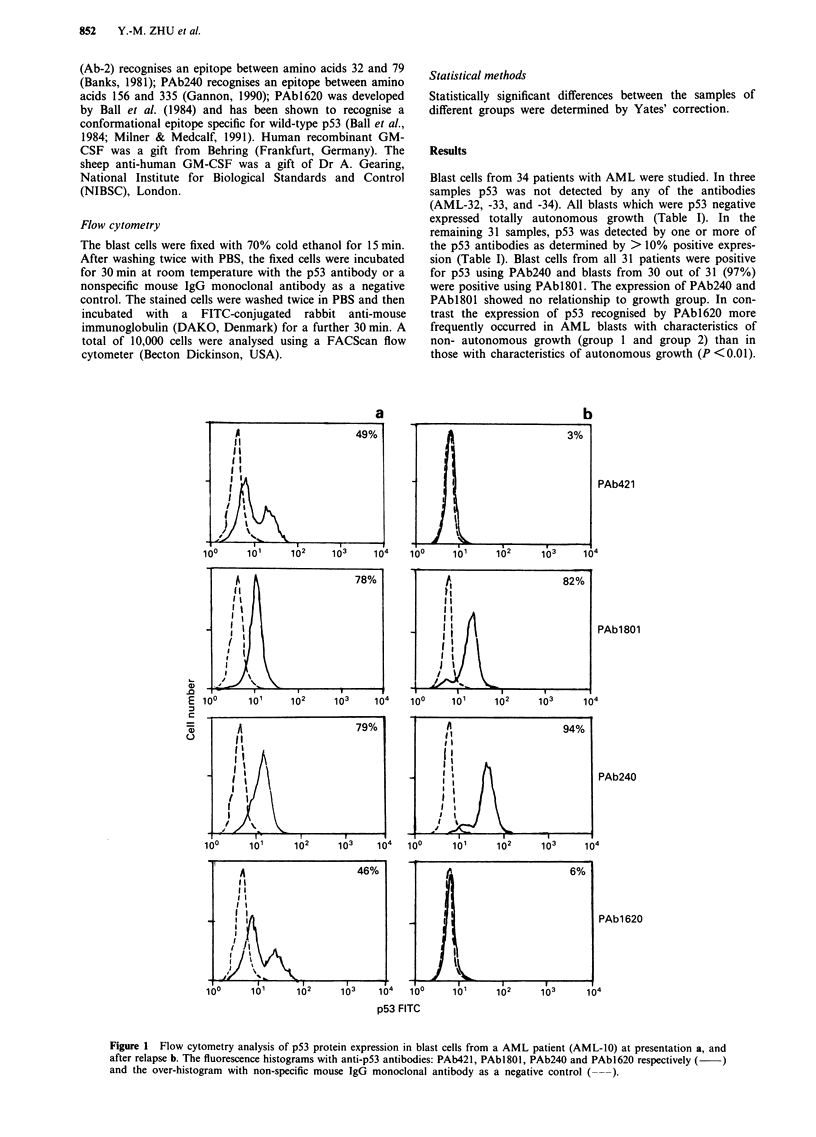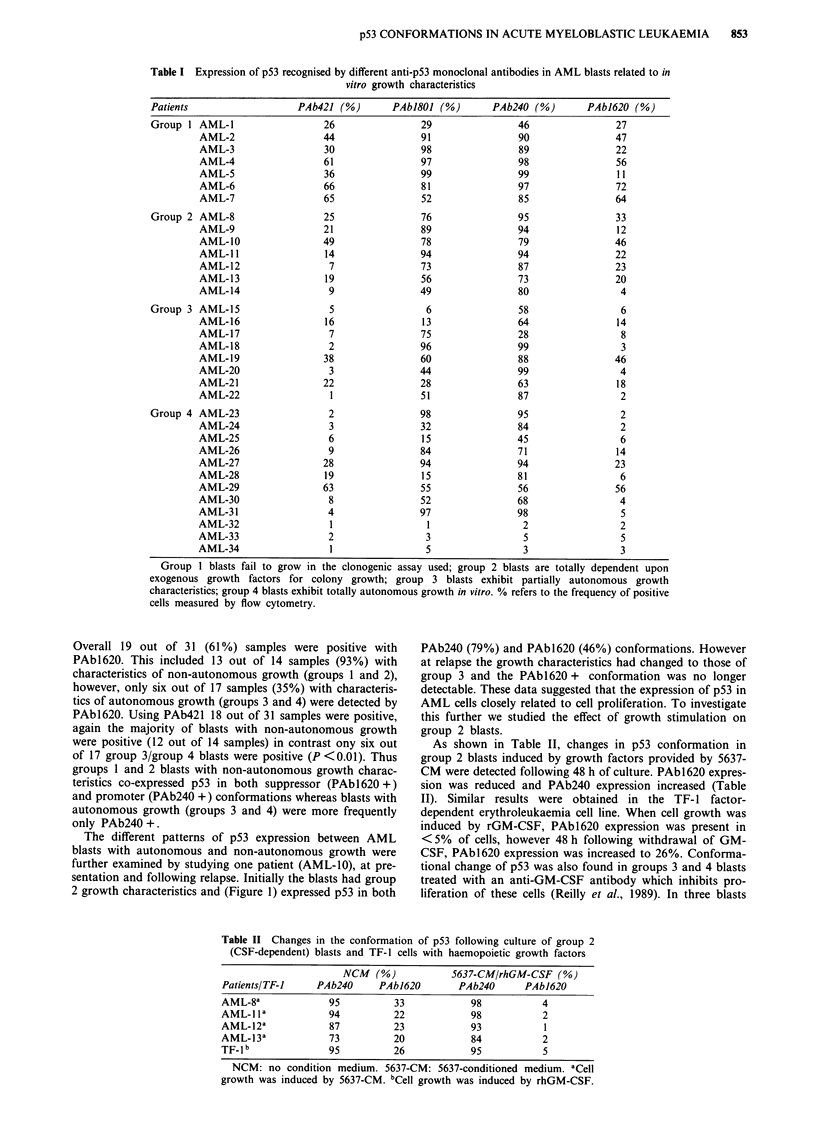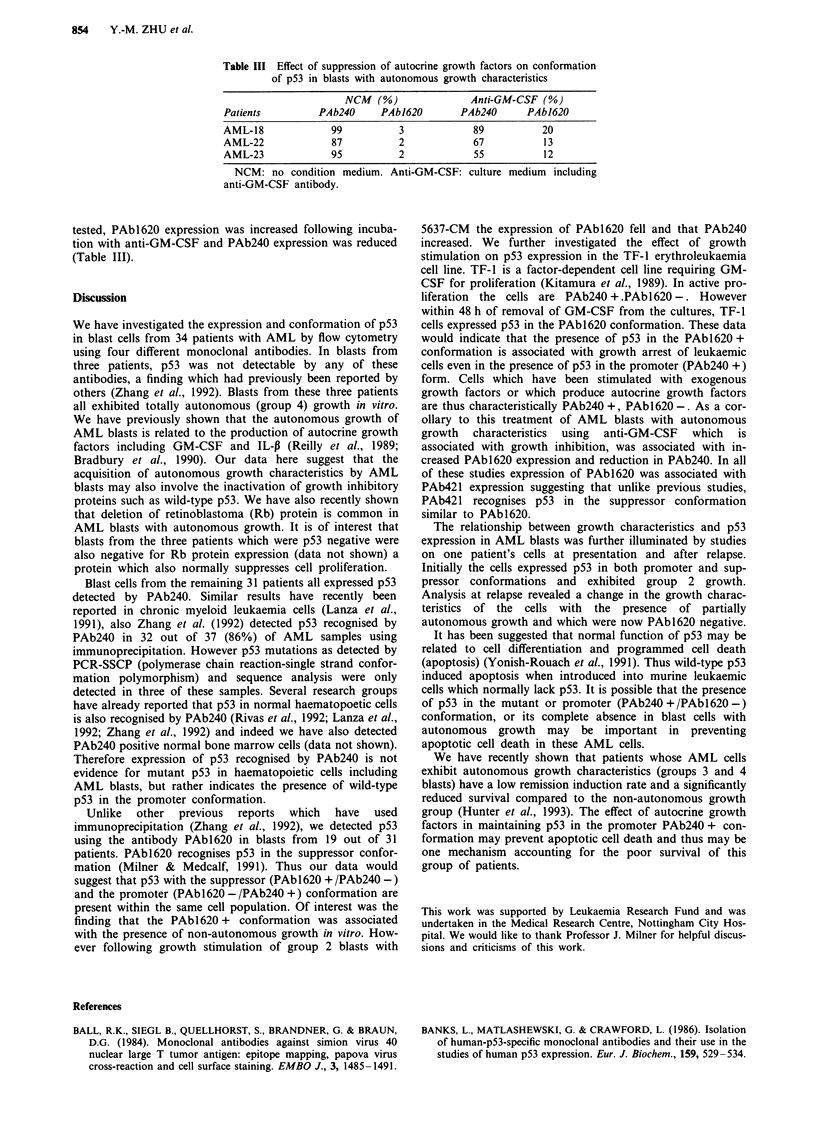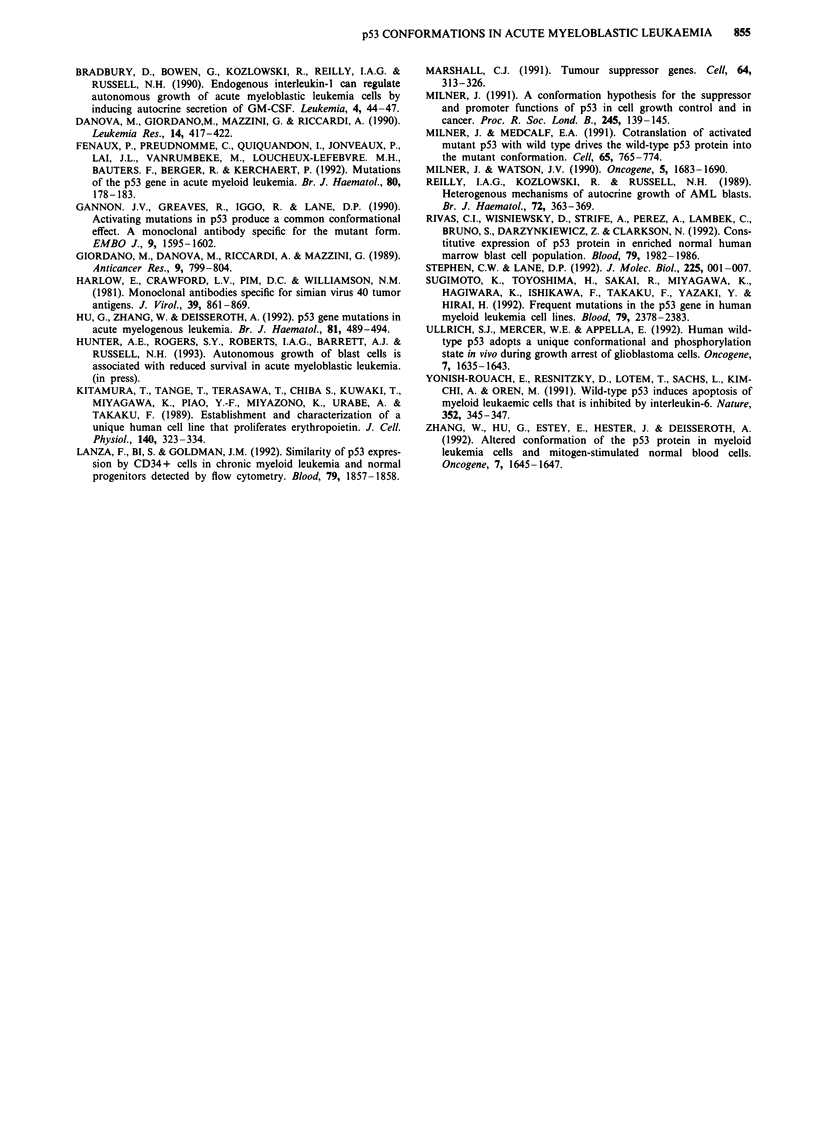# Expression of different conformations of p53 in the blast cells of acute myeloblastic leukaemia is related to in vitro growth characteristics.

**DOI:** 10.1038/bjc.1993.444

**Published:** 1993-11

**Authors:** Y. M. Zhu, D. Bradbury, N. Russell

**Affiliations:** Department of Haematology, Nottingham City Hospital, UK.

## Abstract

Expression of the wild-type p53 gene has an important role in cell differentiation, maturation and apoptosis. Mutation of the p53 gene is associated with tumour development and mutant p53 can promote cell proliferation. Recently wild-type p53 has been demonstrated to exist in two conformational variants: one acting as a suppressor (PAb240-/PAb1620+) and one as a promoter (PAb240+/PAb1620-) of cell proliferation. We have analysed the expression of p53 by flow cytometry in blast cells from 34 patients with acute myeloblastic leukaemia in relationship to the proliferation characteristics of these cells in a clonogenic assay. Blasts from three out of 34 patients did not express p53 using the antibodies: PAb421, PAb1801, PAb240 and PAb1620. The remaining 31 samples expressed p53 detected by PAb240 which recognises mutant p53 and is predicted to recognise wild-type p53 in the promoter conformation. Blasts from 19 out of 31 cells which expressed PAb240 co-expressed PAb1620, expression of PAb1620 was associated with non-autonomous growth in vitro. In contrast, the majority of blasts with the p53 phenotype of PAb240+/PAb1620- or which lacked p53 expression exhibited autonomous growth characteristics in vitro. Furthermore expression of PAb1620 in blasts with autonomous growth cells could be detected following growth inhibition using monoclonal antibodies against autocrine growth factors. Our data demonstrate that in AML cells, p53 conformation is related to the growth characteristics of the cells and is regulated by either exogenous or autocrine haematopoietic growth factors.


Br. J. Cancer (1993), 68, 851-855 © Macmillan Press Ltd., 1993

### Expression of different conformations of p53 in the blast cells of acute myeloblastic leukaemia is related to *in vitro* growth characteristics

Y.-M. Zhu, D. Bradbury & N. Russell

*Department of Haematology, Nottingham City Hospital and University of Nottingham, Nottingham, UK.*

**Summary** Expression of the wild-type p53 gene has an important role in cell differentiation, maturation and apoptosis. Mutation of the p53 gene is associated with tumour development and mutant p53 can promote cell proliferation. Recently wild-type p53 has been demonstrated to exist in two conformational variants: one acting as a suppressor (PAb240 −/PAb1620 +) and one as a promoter (PAb240 +/PAb1620 −) of cell proliferation. We have analysed the expression of p53 by flow cytometry in blast cells from 34 patients with acute myeloblastic leukaemia in relationship to the proliferation characteristics of these cells in a clonogenic assay. Blasts from three out of 34 patients did not express p53 using the antibodies: PAb421, PAb1801, PAb240 and PAb1620. The remaining 31 samples expressed p53 detected by PAb240 which recognises mutant p53 and is predicted to recognise wild-type p53 in the promoter conformation. Blasts from 19 out of 31 cells which expressed PAb240 co-expressed PAb1620, expression of PAb1620 was associated with non-autonomous growth *in vitro*. In contrast, the majority of blasts with the p53 phenotype of PAb240 +/PAb1620 − or which lacked p53 expression exhibited autonomous growth characteristics *in vitro*. Furthermore expression of PAb1620 in blasts with autonomous growth cells could be detected following growth inhibition using monoclonal antibodies against autocrine growth factors. Our data demonstrate that in AML cells, p53 conformation is related to the growth characteristics of the cells and is regulated by either exogenous or autocrine haematopoietic growth factors.

Wild-type p53 gene has properties consistent with it being the product of a tumour suppressor gene. Mutations within highly conserved domains of the p53 gene frequently occur in wide variety of tumour cells including leukaemic cells (Sugimoto *et al.*, 1991; Fenaux *et al.*, 1992; Hu *et al.*, 1992). The products of mutated p53 gene not only lack normal function but also can convert wild-type p53 to a promoter of cell proliferation and tumour progression (Marshall, 1991). Recently evidence has accumulated that wild-type p53 can exist in two different conformations; one with a suppressor effect and one with a promoter effect on cell proliferation (reviewed in Milner, 1991; and Ullrich *et al.*, 1992). These studies have been facilitated by the differential reactivity of the two different conformations with a number of p53 monoclonal antibodies; PAb421, PAb1801, PAb240 and PAb1620. Thus Milner (1991) has proposed that the suppressor form of p53 is PAb1620 +/PAb240 − and that the promoter form is PAb1620 −/PAb240 +, the latter is also the immunophenotype of mutant p53.

The blast cells of acute myeloblastic leukaemia (AML) are a convenient model to study the effect of p53 on cell proliferation. Studies of the *in vitro* growth characteristics of AML blasts have revealed considerable heterogeneity. We have previously classified the growth of AML blasts into four groups depending upon their pattern of growth in a blast cell colony assay. Group 1 cells fail to grow in this system, even when stimulated by exogenous colony-stimulating factors (CSF); group 2 cells form colonies but growth is totally dependent upon exogenous CSFs; group 3 cells exhibit partial autonomous growth which is dependent upon the production of autocrine growth factor but growth can be further stimulated by CSF; finally group 4 cells grow totally autonomously. The autonomous growth of group 3 and group 4 cells was found to be related to the production of autocrine growth factors particularly granulocyte-macrophage CSF (GM-CSF) and interleukin-1β (Reilly *et al.*, 1989; Bradbury *et al.*, 1990). Here we have studied the expression of p53 in AML blasts with different growth characteristics and the effect of stimulation and inhibition of proliferation on the conformation of p53.

Correspondence: N.H. Russell, Department of Haematology, City Hospital, Hucknall Road, Nottingham NG5 1PB, UK.
Received 14 April 1993; and in revised form 24 June 1993.

### Materials and methods

### *AML cells*

Blood samples were obtained at diagnosis from 34 patients with AML and peripheral blood blast count of $>2 \times 10^9\,l^{-1}$. The diagnosis of AML was made using FAB criteria following conventional cytochemical stains and surfaces marker analysis. Mononuclear cells were separated by Ficoll-Hypaque sedimentation and samples were depleted of T cells by Dynabeads M-450 Pan-T (CD-2) (Dynal, Oslo, Norway). TF-1 which is a human factor-dependent erythroleukaemic cell line (Kitamura *et al.*, 1989) was kindly donated by Dr Kitamura (DNAX Research Institute of Molecular and Biology, Inc. Palo Alto, California, USA).

### *Colony assay of AML blasts*

Cells were cultured at $2 \times 10^5$ cells ml$^{-1}$ in 100 µl of Iscove's modified Dulbecco's medium (Flow Labs, Irvine, UK) containing 10% FCS and 0.8% methylcellulose in 100 µl volumes in 96-well microtitre plates as previously described (Reilly *et al.*, 1989). Cultures were plated in triplicate in the presence or absence of a source of colony-stimulating activity provided by medium conditioned by the 5637-human bladder carcinoma cell line which contains GM-CSF, G-CSF and IL-1. Colonies of >20 cells were counted after 5–7 days in culture. For each patient an autostimulatory index (ASI) was calculated which represents the number of colonies/$2 \times 10^4$ cells grown in the absence of 5637-CM divided by the number of colonies/$2 \times 10^4$ in the presence of 5637-CM. Patient cells were classified into groups: group 1 blasts failed to grow in this culture system either autonomously or in response to 5637-CM; group 2 blasts formed colonies, but only in the presence of 5637-CM (ASI < 0.1); group 3 blasts produced significant numbers of colonies (> 10/$2 \times 10^4$ cells without added 5637-CM, but colony growth size and number was further stimulated by 5637-CM (ASI 0.1–0.8); group 4 blasts exhibited totally autonomous growth (ASI > 0.8).

### *Antibodies and growth factors*

Four purified mouse monoclonal antibodies against p53 were obtained from Oncogene Science, Inc. (Uniondale, N.Y., USA). PAb241 (Ab-1) recognises an epitope of p53 located between amino acids 370 and 378 (Harlow, 1981); PAb1801

(Ab-2) recognises an epitope between amino acids 32 and 79 (Banks, 1981); PAb240 recognises an epitope between amino acids 156 and 335 (Gannon, 1990); PAb1620 was developed by Ball *et al.* (1984) and has been shown to recognise a conformational epitope specific for wild-type p53 (Ball *et al.*, 1984; Milner & Medcalf, 1991). Human recombinant GM-CSF was a gift from Behring (Frankfurt, Germany). The sheep anti-human GM-CSF was a gift of Dr A. Gearing, National Institute for Biological Standards and Control (NIBSC), London.

### *Flow cytometry*

The blast cells were fixed with 70% cold ethanol for 15 min. After washing twice with PBS, the fixed cells were incubated for 30 min at room temperature with the p53 antibody or a nonspecific mouse IgG monoclonal antibody as a negative control. The stained cells were washed twice in PBS and then incubated with a FITC-conjugated rabbit anti-mouse immunoglobulin (DAKO, Denmark) for a further 30 min. A total of 10,000 cells were analysed using a FACScan flow cytometer (Becton Dickinson, USA).

### *Statistical methods*

Statistically significant differences between the samples of different groups were determined by Yates' correction.

### Results

Blast cells from 34 patients with AML were studied. In three samples p53 was not detected by any of the antibodies (AML-32, -33, and -34). All blasts which were p53 negative expressed totally autonomous growth (Table I). In the remaining 31 samples, p53 was detected by one or more of the p53 antibodies as determined by $>10\%$ positive expression (Table I). Blast cells from all 31 patients were positive for p53 using PAb240 and blasts from 30 out of 31 (97%) were positive using PAb1801. The expression of PAb240 and PAb1801 showed no relationship to growth group. In contrast the expression of p53 recognised by PAb1620 more frequently occurred in AML blasts with characteristics of non- autonomous growth (group 1 and group 2) than in those with characteristics of autonomous growth ($P<0.01$).

**Figure 1** Flow cytometry analysis of p53 protein expression in blast cells from a AML patient (AML-10) at presentation **a**, and after relapse **b**. The fluorescence histograms with anti-p53 antibodies: PAb421, PAb1801, PAb240 and PAb1620 respectively (——) and the over-histogram with non-specific mouse IgG monoclonal antibody as a negative control (---).

**Table I** Expression of p53 recognised by different anti-p53 monoclonal antibodies in AML blasts related to *in vitro* growth characteristics

| Patients | | PAb421 (%) | PAb1801 (%) | PAb240 (%) | PAb1620 (%) |
|---|---|---|---|---|---|
| Group 1 | AML-1 | 26 | 29 | 46 | 27 |
| | AML-2 | 44 | 91 | 90 | 47 |
| | AML-3 | 30 | 98 | 89 | 22 |
| | AML-4 | 61 | 97 | 98 | 56 |
| | AML-5 | 36 | 99 | 99 | 11 |
| | AML-6 | 66 | 81 | 97 | 72 |
| | AML-7 | 65 | 52 | 85 | 64 |
| Group 2 | AML-8 | 25 | 76 | 95 | 33 |
| | AML-9 | 21 | 89 | 94 | 12 |
| | AML-10 | 49 | 78 | 79 | 46 |
| | AML-11 | 14 | 94 | 94 | 22 |
| | AML-12 | 7 | 73 | 87 | 23 |
| | AML-13 | 19 | 56 | 73 | 20 |
| | AML-14 | 9 | 49 | 80 | 4 |
| Group 3 | AML-15 | 5 | 6 | 58 | 6 |
| | AML-16 | 16 | 13 | 64 | 14 |
| | AML-17 | 7 | 75 | 28 | 8 |
| | AML-18 | 2 | 96 | 99 | 3 |
| | AML-19 | 38 | 60 | 88 | 46 |
| | AML-20 | 3 | 44 | 99 | 4 |
| | AML-21 | 22 | 28 | 63 | 18 |
| | AML-22 | 1 | 51 | 87 | 2 |
| Group 4 | AML-23 | 2 | 98 | 95 | 2 |
| | AML-24 | 3 | 32 | 84 | 2 |
| | AML-25 | 6 | 15 | 45 | 6 |
| | AML-26 | 9 | 84 | 71 | 14 |
| | AML-27 | 28 | 94 | 94 | 23 |
| | AML-28 | 19 | 15 | 81 | 6 |
| | AML-29 | 63 | 55 | 56 | 56 |
| | AML-30 | 8 | 52 | 68 | 4 |
| | AML-31 | 4 | 97 | 98 | 5 |
| | AML-32 | 1 | 1 | 2 | 2 |
| | AML-33 | 2 | 3 | 5 | 5 |
| | AML-34 | 1 | 5 | 3 | 3 |

Group 1 blasts fail to grow in the clonogenic assay used; group 2 blasts are totally dependent upon exogenous growth factors for colony growth; group 3 blasts exhibit partially autonomous growth characteristics; group 4 blasts exhibit totally autonomous growth *in vitro*. % refers to the frequency of positive cells measured by flow cytometry.

Overall 19 out of 31 (61%) samples were positive with PAb1620. This included 13 out of 14 samples (93%) with characteristics of non-autonomous growth (groups 1 and 2), however, only six out of 17 samples (35%) with characteristics of autonomous growth (groups 3 and 4) were detected by PAb1620. Using PAb421 18 out of 31 samples were positive, again the majority of blasts with non-autonomous growth were positive (12 out of 14 samples) in contrast ony six out of 17 group 3/group 4 blasts were positive ($P < 0.01$). Thus groups 1 and 2 blasts with non-autonomous growth characteristics co-expressed p53 in both suppressor (PAb1620 +) and promoter (PAb240 +) conformations whereas blasts with autonomous growth (groups 3 and 4) were more frequently only PAb240 +.

The different patterns of p53 expression between AML blasts with autonomous and non-autonomous growth were further examined by studying one patient (AML-10), at presentation and following relapse. Initially the blasts had group 2 growth characteristics and (Figure 1) expressed p53 in both PAb240 (79%) and PAb1620 (46%) conformations. However at relapse the growth characteristics had changed to those of group 3 and the PAb1620 + conformation was no longer detectable. These data suggested that the expression of p53 in AML cells closely related to cell proliferation. To investigate this further we studied the effect of growth stimulation on group 2 blasts.

As shown in Table II, changes in p53 conformation in group 2 blasts induced by growth factors provided by 5637-CM were detected following 48 h of culture. PAb1620 expression was reduced and PAb240 expression increased (Table II). Similar results were obtained in the TF-1 factor-dependent erythroleukaemia cell line. When cell growth was induced by rGM-CSF, PAb1620 expression was present in < 5% of cells, however 48 h following withdrawal of GM-CSF, PAb1620 expression was increased to 26%. Conformational change of p53 was also found in groups 3 and 4 blasts treated with an anti-GM-CSF antibody which inhibits proliferation of these cells (Reilly *et al.*, 1989). In three blasts

**Table II** Changes in the conformation of p53 following culture of group 2 (CSF-dependent) blasts and TF-1 cells with haemopoietic growth factors

| | NCM (%) | | 5637-CM/rhGM-CSF (%) | |
|---|---|---|---|---|
| Patients/TF-1 | PAb240 | PAb1620 | PAb240 | PAb1620 |
| AML-8[a] | 95 | 33 | 98 | 4 |
| AML-11[a] | 94 | 22 | 98 | 2 |
| AML-12[a] | 87 | 23 | 93 | 1 |
| AML-13[a] | 73 | 20 | 84 | 2 |
| TF-1[b] | 95 | 26 | 95 | 5 |

NCM: no condition medium. 5637-CM: 5637-conditioned medium. [a]Cell growth was induced by 5637-CM. [b]Cell growth was induced by rhGM-CSF.

**Table III** Effect of suppression of autocrine growth factors on conformation of p53 in blasts with autonomous growth characteristics

| Patients | NCM (%) PAb240 | NCM (%) PAb1620 | Anti-GM-CSF (%) PAb240 | Anti-GM-CSF (%) PAb1620 |
|---|---|---|---|---|
| AML-18 | 99 | 3 | 89 | 20 |
| AML-22 | 87 | 2 | 67 | 13 |
| AML-23 | 95 | 2 | 55 | 12 |

NCM: no condition medium. Anti-GM-CSF: culture medium including anti-GM-CSF antibody.

tested, PAb1620 expression was increased following incubation with anti-GM-CSF and PAb240 expression was reduced (Table III).

### Discussion

We have investigated the expression and conformation of p53 in blast cells from 34 patients with AML by flow cytometry using four different monoclonal antibodies. In blasts from three patients, p53 was not detectable by any of these antibodies, a finding which had previously been reported by others (Zhang *et al.*, 1992). Blasts from these three patients all exhibited totally autonomous (group 4) growth *in vitro*. We have previously shown that the autonomous growth of AML blasts is related to the production of autocrine growth factors including GM-CSF and IL-β (Reilly *et al.*, 1989; Bradbury *et al.*, 1990). Our data here suggest that the acquisition of autonomous growth characteristics by AML blasts may also involve the inactivation of growth inhibitory proteins such as wild-type p53. We have also recently shown that deletion of retinoblastoma (Rb) protein is common in AML blasts with autonomous growth. It is of interest that blasts from the three patients which were p53 negative were also negative for Rb protein expression (data not shown) a protein which also normally suppresses cell proliferation.

Blast cells from the remaining 31 patients all expressed p53 detected by PAb240. Similar results have recently been reported in chronic myeloid leukaemia cells (Lanza *et al.*, 1991), also Zhang *et al.* (1992) detected p53 recognised by PAb240 in 32 out of 37 (86%) of AML samples using immunoprecipitation. However p53 mutations as detected by PCR-SSCP (polymerase chain reaction-single strand conformation polymorphism) and sequence analysis were only detected in three of these samples. Several research groups have already reported that p53 in normal haematopoetic cells is also recognised by PAb240 (Rivas *et al.*, 1992; Lanza *et al.*, 1992; Zhang *et al.*, 1992) and indeed we have also detected PAb240 positive normal bone marrow cells (data not shown). Therefore expression of p53 recognised by PAb240 is not evidence for mutant p53 in haematopoietic cells including AML blasts, but rather indicates the presence of wild-type p53 in the promoter conformation.

Unlike other previous reports which have used immunoprecipitation (Zhang *et al.*, 1992), we detected p53 using the antibody PAb1620 in blasts from 19 out of 31 patients. PAb1620 recognises p53 in the suppressor conformation (Milner & Medcalf, 1991). Thus our data would suggest that p53 with the suppressor (PAb1620 +/PAb240 −) and the promoter (PAb1620 −/PAb240 +) conformation are present within the same cell population. Of interest was the finding that the PAb1620 + conformation was associated with the presence of non-autonomous growth *in vitro*. However following growth stimulation of group 2 blasts with 5637-CM the expression of PAb1620 fell and that PAb240 increased. We further investigated the effect of growth stimulation on p53 expression in the TF-1 erythroleukaemia cell line. TF-1 is a factor-dependent cell line requiring GM-CSF for proliferation (Kitamura *et al.*, 1989). In active proliferation the cells are PAb240 +.PAb1620 −. However within 48 h of removal of GM-CSF from the cultures, TF-1 cells expressed p53 in the PAb1620 conformation. These data would indicate that the presence of p53 in the PAb1620 + conformation is associated with growth arrest of leukaemic cells even in the presence of p53 in the promoter (PAb240 +) form. Cells which have been stimulated with exogenous growth factors or which produce autocrine growth factors are thus characteristically PAb240 +, PAb1620 −. As a corollary to this treatment of AML blasts with autonomous growth characteristics using anti-GM-CSF which is associated with growth inhibition, was associated with increased PAb1620 expression and reduction in PAb240. In all of these studies expression of PAb1620 was associated with PAb421 expression suggesting that unlike previous studies, PAb421 recognises p53 in the suppressor conformation similar to PAb1620.

The relationship between growth characteristics and p53 expression in AML blasts was further illuminated by studies on one patient's cells at presentation and after relapse. Initially the cells expressed p53 in both promoter and suppressor conformations and exhibited group 2 growth. Analysis at relapse revealed a change in the growth characteristics of the cells with the presence of partially autonomous growth and which were now PAb1620 negative.

It has been suggested that normal function of p53 may be related to cell differentiation and programmed cell death (apoptosis) (Yonish-Rouach *et al.*, 1991). Thus wild-type p53 induced apoptosis when introduced into murine leukaemic cells which normally lack p53. It is possible that the presence of p53 in the mutant or promoter (PAb240 +/PAb1620 −) conformation, or its complete absence in blast cells with autonomous growth may be important in preventing apoptotic cell death in these AML cells.

We have recently shown that patients whose AML cells exhibit autonomous growth characteristics (groups 3 and 4 blasts) have a low remission induction rate and a significantly reduced survival compared to the non-autonomous growth group (Hunter *et al.*, 1993). The effect of autocrine growth factors in maintaining p53 in the promoter PAb240 + conformation may prevent apoptotic cell death and thus may be one mechanism accounting for the poor survival of this group of patients.

This work was supported by Leukaemia Research Fund and was undertaken in the Medical Research Centre, Nottingham City Hospital. We would like to thank Professor J. Milner for helpful discussions and criticisms of this work.

### References

BALL, R.K., SIEGL B., QUELLHORST, S., BRANDNER, G. & BRAUN, D.G. (1984). Monoclonal antibodies against simion virus 40 nuclear large T tumor antigen: epitope mapping, papova virus cross-reaction and cell surface staining. *EMBO J.*, **3**, 1485–1491.

BANKS, L., MATLASHEWSKI, G. & CRAWFORD, L. (1986). Isolation of human-p53-specific monoclonal antibodies and their use in the studies of human p53 expression. *Eur. J. Biochem.*, **159**, 529–534.

BRADBURY, D., BOWEN, G., KOZLOWSKI, R., REILLY, I.A.G. & RUSSELL, N.H. (1990). Endogenous interleukin-1 can regulate autonomous growth of acute myeloblastic leukemia cells by inducing autocrine secretion of GM-CSF. *Leukemia*, **4**, 44–47.

DANOVA, M., GIORDANO,M., MAZZINI, G. & RICCARDI, A. (1990). *Leukemia Res.*, **14**, 417–422.

FENAUX, P., PREUDNOMME, C., QUIQUANDON, I., JONVEAUX, P., LAI, J.L., VANRUMBEKE, M., LOUCHEUX-LEFEBVRE. M.H., BAUTERS. F., BERGER, R. & KERCHAERT, P. (1992). Mutations of the p53 gene in acute myeloid leukemia. *Br. J. Haematol.*, **80**, 178–183.

GANNON. J.V., GREAVES, R., IGGO, R. & LANE, D.P. (1990). Activating mutations in p53 produce a common conformational effect. A monoclonal antibody specific for the mutant form. *EMBO J.*, **9**, 1595–1602.

GIORDANO, M., DANOVA, M., RICCARDI, A. & MAZZINI, G. (1989). *Anticancer Res.*, **9**, 799–804.

HARLOW, E., CRAWFORD, L.V., PIM, D.C. & WILLIAMSON, N.M. (1981). Monoclonal antibodies specific for simian virus 40 tumor antigens. *J. Virol.*, **39**, 861–869.

HU, G., ZHANG, W. & DEISSEROTH, A. (1992). p53 gene mutations in acute myelogenous leukemia. *Br. J. Haematol.*, **81**, 489–494.

HUNTER, A.E., ROGERS, S.Y., ROBERTS, I.A.G., BARRETT, A.J. & RUSSELL, N.H. (1993). Autonomous growth of blast cells is associated with reduced survival in acute myeloblastic leukemia. (in press).

KITAMURA, T., TANGE, T., TERASAWA, T., CHIBA S., KUWAKI, T., MIYAGAWA, K., PIAO, Y.-F., MIYAZONO, K., URABE, A. & TAKAKU, F. (1989). Establishment and characterization of a unique human cell line that proliferates erythropoietin. *J. Cell. Physiol.*, **140**, 323–334.

LANZA, F., BI, S. & GOLDMAN, J.M. (1992). Similarity of p53 expression by CD34+ cells in chronic myeloid leukemia and normal progenitors detected by flow cytometry. *Blood*, **79**, 1857–1858.

MARSHALL, C.J. (1991). Tumour suppressor genes. *Cell*, **64**, 313–326.

MILNER, J. (1991). A conformation hypothesis for the suppressor and promoter functions of p53 in cell growth control and in cancer. *Proc. R. Soc. Lond. B.*, **245**, 139–145.

MILNER, J. & MEDCALF, E.A. (1991). Cotranslation of activated mutant p53 with wild type drives the wild-type p53 protein into the mutant conformation. *Cell*, **65**, 765–774.

MILNER, J. & WATSON, J.V. (1990). *Oncogene*, **5**, 1683–1690.

REILLY, I.A.G., KOZLOWSKI, R. & RUSSELL, N.H. (1989). Heterogenous mechanisms of autocrine growth of AML blasts. *Br. J. Haematol.*, **72**, 363–369.

RIVAS, C.I., WISNIEWSKY, D., STRIFE, A., PEREZ, A., LAMBEK, C., BRUNO, S., DARZYNKIEWICZ, Z. & CLARKSON, N. (1992). Constitutive expression of p53 protein in enriched normal human marrow blast cell population. *Blood*, **79**, 1982–1986.

STEPHEN, C.W. & LANE, D.P. (1992). *J. Molec. Biol.*, **225**, 001–007.

SUGIMOTO, K., TOYOSHIMA, H., SAKAI, R., MIYAGAWA, K., HAGIWARA, K., ISHIKAWA, F., TAKAKU, F., YAZAKI, Y. & HIRAI, H. (1992). Frequent mutations in the p53 gene in human myeloid leukemia cell lines. *Blood*, **79**, 2378–2383.

ULLRICH, S.J., MERCER, W.E. & APPELLA, E. (1992). Human wild-type p53 adopts a unique conformational and phosphorylation state *in vivo* during growth arrest of glioblastoma cells. *Oncogene*, **7**, 1635–1643.

YONISH-ROUACH, E., RESNITZKY, D., LOTEM, T., SACHS, L., KIMCHI, A. & OREN, M. (1991). Wild-type p53 induces apoptosis of myeloid leukaemic cells that is inhibited by interleukin-6. *Nature*, **352**, 345–347.

ZHANG, W., HU, G., ESTEY, E., HESTER, J. & DEISSEROTH, A. (1992). Altered conformation of the p53 protein in myeloid leukemia cells and mitogen-stimulated normal blood cells. *Oncogene*, **7**, 1645–1647.